# Methicillin-resistant *Staphylococcus aureus* eradication in cystic fibrosis patients: A randomized multicenter study

**DOI:** 10.1371/journal.pone.0213497

**Published:** 2019-03-22

**Authors:** Daniela Dolce, Stella Neri, Laura Grisotto, Silvia Campana, Novella Ravenni, Francesca Miselli, Erica Camera, Lucia Zavataro, Cesare Braggion, Ersilia V. Fiscarelli, Vincenzina Lucidi, Lisa Cariani, Daniela Girelli, Nadia Faelli, Carla Colombo, Cristina Lucanto, Mariangela Lombardo, Giuseppe Magazzù, Antonella Tosco, Valeria Raia, Serena Manara, Edoardo Pasolli, Federica Armanini, Nicola Segata, Annibale Biggeri, Giovanni Taccetti

**Affiliations:** 1 Cystic Fibrosis Center, Anna Meyer Children's University Hospital, University of Florence, Florence, Italy; 2 Department of Health Sciences, University of Florence, Florence, Italy; 3 Department of Statistics, Computer Science and Applications "G. Parenti", University of Florence, Florence, Italy; 4 Cystic Fibrosis Microbiology and Cystic Fibrosis Center, Children's Hospital and Research Institute Bambino Gesù, Rome, Italy; 5 Laboratory for Cystic Fibrosis Microbiology, Fondazione IRCCS, Ca' Granda—Ospedale Maggiore Policlinico, Milan, Italy; 6 Cystic Fibrosis Center, University of Milan, Fondazione IRCCS, Ca' Granda—Ospedale Maggiore Policlinico, Milan, Italy; 7 Cystic Fibrosis and Pediatric Gastroenterology Unit, University of Messina, Messina, Italy; 8 Department of Translational Medical Sciences, Pediatric Cystic Fibrosis Center, Federico II University, Naples, Italy; 9 Center for Integrative Biology, University of Trento, Trento, Italy; Public Library of Science, UNITED KINGDOM

## Abstract

**Background:**

Few studies, based on a limited number of patients using non-uniform therapeutic protocols, have analyzed Methicillin-resistant Staphylococcus aureus (MRSA) eradication.

**Methods:**

In a randomized multicenter trial conducted on patients with new-onset MRSA infection we evaluated the efficacy of an early eradication treatment (arm A) compared with an observational group (B). Arm A received oral rifampicin and trimethoprim/sulfamethoxazole (21 days). Patients’ microbiological status, FEV_1_, BMI, pulmonary exacerbations and use of antibiotics were assessed.

**Results:**

Sixty-one patients were randomized. Twenty-nine (47.5%) patients were assigned to active arm A and 32 (52.5%) patients to observational arm B. Twenty-nine (47.5%) patients, 10 patients in arm A and 19 in arm B, dropped out of the study. At 6 months MRSA was eradicated in 12 (63.2%) out of 19 patients in arm A while spontaneous clearance was observed in 5 (38.5%) out of 13 patients in arm B. A per-protocol analysis showed a 24.7% difference in the proportion of MRSA clearance between the two groups (z = 1.37, P(Z>z) = 0.08). Twenty-seven patients, 15 (78.9%) out of 19 in arm A and 12 (92.3%) out of 13 in arm B, were able to perform spirometry. The mean (±SD) FEV_1_ change from baseline was 7.13% (±14.92) in arm A and -1.16% (±5.25) in arm B (p = 0.08). In the same period the BMI change (mean ±SD) from baseline was 0.54 (±1.33) kg/m^2^ in arm A and -0.38 (±1.56) kg/m^2^ in arm B (p = 0.08). At 6 months no statistically significant differences regarding the number of pulmonary exacerbations, days spent in hospital and use of antibiotics were observed between the two arms.

**Conclusions:**

Although the statistical power of the study is limited, we found a 24.7% higher clearance of MRSA in the active arm than in the observational arm at 6 months. Patients in the active arm A also had favorable FEV_1_ and BMI tendencies.

## Introduction

Pulmonary infection is the principal characteristic of cystic fibrosis (CF) and the main cause of morbidity and mortality [1–4]. The major pathogens responsible for respiratory tract infections in CF patients are *Staphylococcus aureus* and *Pseudomonas aeruginosa* [1,2]. Methicillin-resistant *Staphylococcus aureus* (MRSA) infection is a matter of concern since persistent infection due to this bacterium is associated with an increased rate of decline in lung function and higher mortality [2–4].

Only a limited number of studies have analyzed the pros and cons of early MRSA eradication, and all reported experiences are based on a limited number of patients, on the use of non-uniform therapeutic protocols and on heterogeneous topical decolonization practices [5–10]. Furthermore, the definitions of eradication used for MRSA infection do not fully satisfy the definition of eradication used in clinical practice for other pathogens such as *P*. *aeruginosa*, which is usually based on 3 negative cultures in a 6-month period [11–13]. Moreover, the long-term risk of the emergence of new pathogens due to treatment has only been partly evaluated. In the absence of a gold standard treatment for new-onset MRSA infection [5–10], we hypothesized that an early eradication protocol enacted at the time of a first or new MRSA infection is a more effective way to clear this pathogen in comparison to observation alone.

## Aims of the study

The primary aim of this open-label, randomized, multicenter, parallel-group study was to evaluate, on a sample of clinically stable CF patients, the efficacy of an eradication protocol against new-onset MRSA infection in comparison to observation alone.

The secondary aims of this study were:

1. to assess the change in forced expiratory volume in one second (FEV_1_) and body mass index (BMI) in patients in the active arm (A) and in the observation arm (B) during a time span of 6 months;
2. to determine the existence of any differences between the 2 arms in regard to the period in which the patient remains MRSA-free;
3. to determine if the eradication treatment is associated with an increasing risk of emergence of particular pathogens (*Burkholderia cepacia* complex and other non-fermentative Gram-negative bacteria) in the respiratory tract;
4. to assess the number of pulmonary exacerbations and hospitalizations, the days of total (oral, inhaled and intravenous) antibiotic usage, in the 2 arms during a time span of 6 months;
5. to evaluate the antibiotic susceptibility and molecular characteristics of MRSA strains isolated from the airways of CF patients experiencing new-onset MRSA infection.

## Materials and methods

### Centers

Five CF Referral Centers (Florence, Rome, Milan, Messina and Naples), established by Italian law [14], participated in this trial. The study received ethical approval from the Ethics Committees of Meyer Hospital (Florence), Institute Bambino Gesù (Rome), Milan, Messina and Naples and written patient consent was obtained. The trial was registered as Eudract (EU Clinical Trials Register) number 2013-000219-25.

### Participants

Patients in regular clinical and microbiological follow-up [15] were considered eligible if more than 4 years old and experiencing new-onset MRSA infection. New-onset MRSA infection was considered baseline and was defined as either a first isolation of MRSA from the airways of the CF patient or a new MRSA isolation after a clearance period of 12 months (after performance of 4 negative cultures).

CF diagnosis was based on clinical features of the disease, ≥ 60 mmol/L concentration of chloride in sweat and/or the presence of two CF-causing mutations [16].

Patients were excluded from the study on the basis of the following criteria:

- Respiratory exacerbation [17] at the time of randomization
- History of hypersensitivity to or adverse reaction to antibiotics used in the intervention of the study.
- Liver cirrhosis or abnormal liver function test results at study entry (defined as ALT and/or AST levels more than twice the upper limit of the normal range)
- Abnormal kidney function at study entry (defined as a serum creatinine level >1.5 times the upper limit normal for the participant’s age)
- Pregnancy
- Lung/liver transplantation
- Contemporaneous use of any investigational drug
- MRSA resistance to both antibiotics, trimethoprim/sulfamethoxazole (TMP/SMX) and rifampicin

Written informed consent was obtained from all participants in the study. Parents gave their consent for minors.

### Randomization

Between July 18^th^, 2013, and April 12^th^, 2016, 61 CF patients with a first/new MRSA infection were randomly assigned to the active arm (A) or observational arm (B). A balanced randomization sequence with permuted blocks of size 4 was created using statistical software. Randomization assignment, performed at the coordinator Center (Meyer Hospital), was organized by e-mail. Patients, allocated 1:1, were enrolled at their own CF Center. The people involved in randomization and in the treatment assignments were kept completely separate [18].

### Procedures

Patients randomized to the active arm (A) were treated with the following antibiotic regimen:

- Oral rifampicin 15 mg/kg/day in 2 daily doses (maximum daily dose 600 mg) for 21 days
- Oral TMP-SMX 8–40 mg/kg/day in 2 daily doses (maximum daily dose 320/1600 mg) for 21 days
- 2% nasal mupirocin–each nostril 3 times daily for 5 days

In case of antibiotic resistance, an alternative approach was planned: patients over 8 years of age were treated with rifampicin and minocycline when MRSA was resistant to TMP/SMX, or with TMP/SMX and minocycline (pediatric dose: 2 mg/kg orally twice daily for 21 days, adult dose: 100 mg orally twice daily for 21 days) when MRSA was resistant to rifampicin.

Physicians of the respective Centers managed the patients’ clinical course according to standards of care [15]. Treatment was suspended in cases of adverse effects or pulmonary exacerbation [17].

Treatment costs were covered by the Italian National Health Service at no charge to the patients [14]. FEV_1_ values were measured according to ATS-ERS standards [19].

Patients’ microbiological status was determined according to the European CF Registry definitions at the time of new-onset MRSA infection [1].

Microbiological analyses were performed following published literature. Antibiotic susceptibility was evaluated using the VITEK2 (bioMérieux) automated system and EUCAST clinical breakpoints were used as interpretation criteria [*www*.*eucast*.*org/clinical_breakpoints/*]. DNA extractions were performed for each isolate. To determine the potential virulence of MRSA strains, a specific PCR assay for the presence of the Panton-Valentine Leukocidin (PVL) gene was performed [20]. The *mecA* gene and other loci of the SCC*mec* cassette were analyzed using different multiplex PCR [21].

Sequence typing and *spa*-typing were performed by whole genome sequencing and analyzed using MetaMLST [22] and the DNAGear software [23] respectively.

### Outcomes

The primary outcome was MRSA eradication, defined as the patient having 3 successive negative cultures in 6 months according to the United Kingdom CF Trust criteria [24]. During this same 6-month period, we also assessed the patients’ FEV_1_ change, nutritional status (BMI), pulmonary exacerbations and antibiotic use. Having received antibiotics potentially active against MRSA during the follow-up was considered a cause of drop-out.

Results of cultures and clinical records were used to assess secondary aims.

### Statistical analyses

This trial was designed by calculating the sample size as a balance between statistical considerations [18] and epidemiological experience of MRSA infection in Italy [25]. We hypothesized that MRSA eradication would occur in 75% of cases in the active arm and spontaneous clearance in 50% of the observation group. Using a one-tailed test (which means that only an effect in the expected direction is interpreted), and having set the alpha (type I) error at 0.05 and the beta (type II) error at 30%, we planned to enroll 60 patients (30 per arm) in a 2-year trial to reach statistical significance. A 25% greater rate of eradication in arm A in comparison with arm B was considered clinically relevant. Data were independent, with one observation per participant [18].

The results of the study were reviewed and evaluated by the Data Safety Monitoring Board. Since recruitment was behind schedule, this Board agreed on an extension of the recruitment period by one year in all the participating Centers. The necessity of administering antibiotics during the 6-month follow-up period (in order to satisfy the definition of eradication) led us to perform a per-protocol analysis because an intention-to-treat analysis would not have provided an appropriate interpretation of the data [18]. The primary outcome was evaluated using a two sample test of proportion. The percentage of MRSA free patients was assessed during the follow-up at 60, 120 and 180 days; 95% confidence intervals (CI) were calculated using exact likelihood ratios.

Regarding secondary aims, quantitative variables were expressed as mean ± SD. The differences between the 2 arms regarding continuous variables were assessed using Student’s t-test. Level of significance was set to 5%, two-tailed, when not otherwise specified.

We conducted all statistical analyses using STATA version 13.0 (StataCorp.2013. Stata Statistical Software: Release 13. College Station, TX: StataCorp LP).

## Results

**Fig 1:**
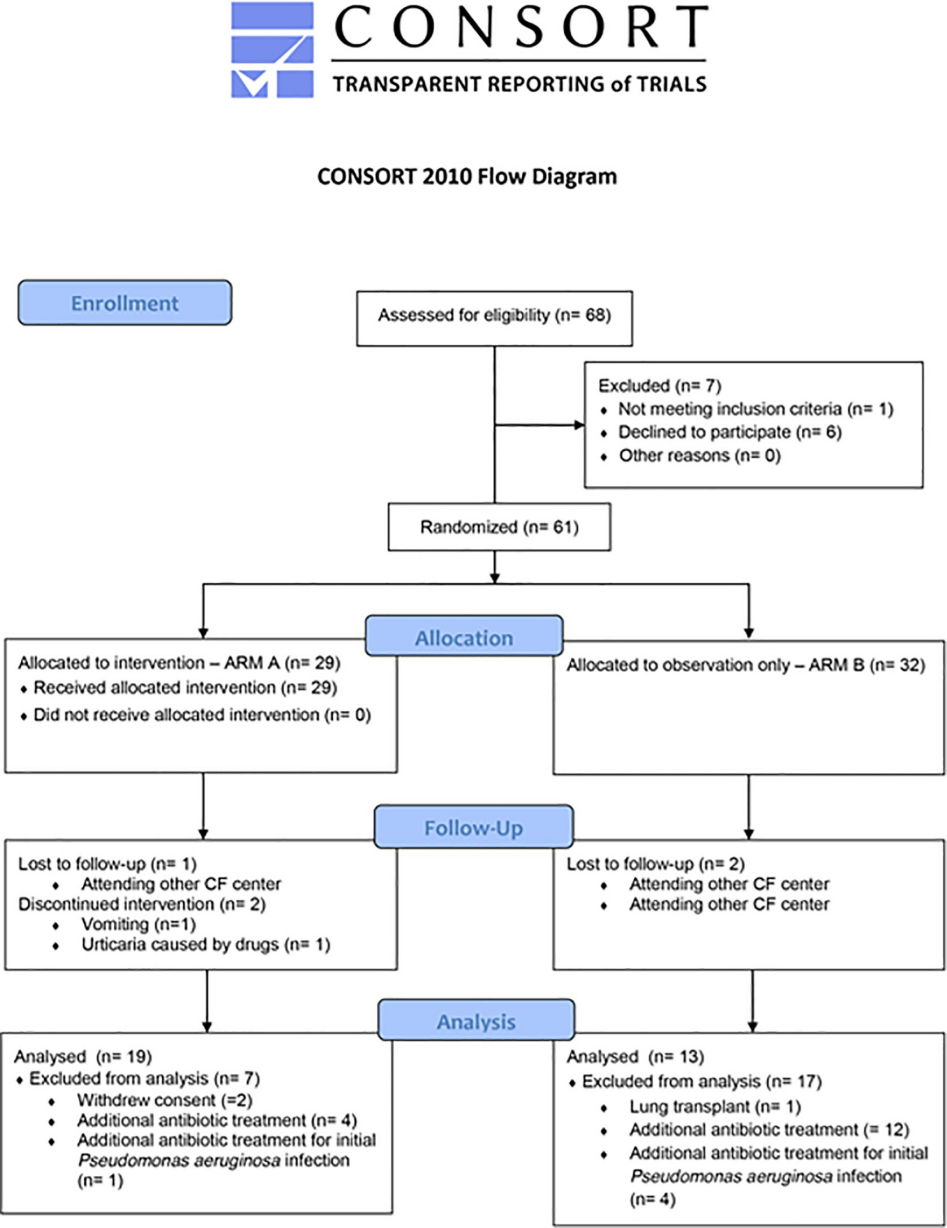
Trial profile.

Fig 1 shows the trial profile. Sixty-eight patients were assessed for eligibility from February 1, 2013 to April 30, 2016. Sixty-one (89.7%) patients were randomized with 29 (47.5%) patients being assigned to arm A and 32 (52.5%) to arm B.

The oral rifampicin/TMP-SMX protocol was used in 25 (86.2%) out of 29 patients in the active arm, and an alternative approach was used in 4 (13.8%) patients (2 patients were treated with both TMP-SMX/minocycline due to rifampicin resistance and 2 others with both rifampicin/minocycline due to suspected TMP-SMX allergy or side effects). The time (mean ± SD) from new-onset MRSA infection diagnosis to the time of treatment initiation was 27 ± 32 days.

Twenty-nine (47.5%) out of 61 randomized patients, 10 patients in arm A and 19 in arm B, dropped out of the study. Causes of drop-out are shown in Fig 1. The main cause of drop-out was the administration of further antibiotic treatment due to variations in the patient’s clinical condition during follow-up in 4 (40%) out of 10 patients in arm A and 12 (63.2%) out of 19 in arm B.

Table 1 shows patients’ clinical characteristics.

**Table 1:** Patients’ characteristics.

	Arm A (active arm)	Arm B (observational arm)
Number of patients	(n = 29)	(n = 32)
**Age in years**		
Mean ± SD Median (range)	16.97 ± 12.19 14.5 (2.6–45.2)	17.19 ± 12.74 13.6 (2.4–50.7)
**Gender**		
Male	20 (69.0%)	15 (46.9%)
Female	9 (31.0%)	17 (53.1%)
**CFTR genotype**		
F508del homozygotes	7 (24.1%)	6 (18.8%)
F508del heterozygotes	13 (44.9%)	16 (50.0%)
Other genotype	9 (31.0%)	10 (31.2%)
**Anthropometrics (**mean ± SD)		
Weight (kgs)	45.15 ± 21.85	41.84 ± 20.09
Height (cm)	146.64 ± 27.02	142.95 ± 26.48
BMI (Kg/m^2^)	19.18 ± 3.92	18.75 ± 4.11
**Spirometry**		
FEV_1_ (L)	2.20 ± 0.97	2.05 ± 0.87
FEV_1_ (% of predicted)	78.13 ± 24.70	81.95 ± 27.31
**FEV**_**1**_ **groups**		
≥ 70	16 (55.2%)	21 (65.6%)
< 70	8 (27.6%)	7 (21.9%)
Unable to perform spirometry	5 (17.2%)	4 (12.5%)
**First MRSA infection**	26 (89.7%)	29 (90.6%)
**Previously infected**	3 (10.3%)	3 (9.4%)
**Sampling methods**		
Throat swabs	7 (24.1%)	5 (15.6%)
Sputum	22 (75.9%)	27 (84.4%)
**Patients’ microbiological status for other pathogens at baseline**		
**Methicillin-sensitive *Staphylococcus aureus*** (MSSA)		
Positive	10 (34.5%)	12 (37.5%)
Negative	19 (65.5%)	20 (62.5%)
***P*. *aeruginosa***		
Positive [chronic]	12 (41.4%) [6]	17 (53.1%) [4]
Negative	17 (58.6%)	15 (46.9%)
***A*. *xylosoxidans***		
Positive	1 (3.4%)	1 (3.1%)
Negative	28 (96.6%)	31 (96.9%)
***S*. *maltophilia***		
Positive	1 (3.4%)	4 (12.5%)
Negative	28 (96.6%)	28 (87.5%)
***Aspergillus spp***		
Positive	0 (0%)	5 (15.6%)
Negative	29 (100%)	27 (84.4%)
**Other**		
	6 (20.7%)	11 (34.4%)
	4 with *H*. *influenzae*	9 with *H*. *influenzae*
	2 with *B*. *gladioli*	2 with nontuberculous mycobacteria

Fifty-two (85.2%) out of 61 participants, 24 patients in arm A and 28 in arm B, were able to perform spirometry (≥5 years of age).

MRSA was firstly isolated in 55 (90.2%) out of 61 patients included in this trial, 26 (89.7%) in arm A and 29 (90.6%) in arm B, while MRSA was previously isolated in 6 (9.8%) patients, 3 (10.3%) in arm A and 3 (9.4%) in arm B. No statistically significant difference in first and previously MRSA infected patients was observed between the 2 arms (p = 1). In those patients who had previous MRSA infection the mean (±SD) time from the previous isolation was 741±506.89 days in arm A and 1630±1265.40 days in arm B (p = 0.32).

Microbiological cultures were performed on specimens collected by throat swabs in 12 (19.7%) out of 61 patients, 7 (24.1%) out of 29 patients in arm A and 5 (15.6%) out of 32 patients in arm B (Table 1). Sputum cultures were performed in 49 (80.3%) patients, 22 (75.9%) out of 29 patients in arm A and 27 (84.4%) out of 32 patients in arm B. No statistically significant difference regarding sampling methods was observed between the 2 arms (p = 0.6).

No substantial differences regarding chronic/intermittent co-infections with other causative pathogens were observed between the groups.

Five patients from whom *Aspergillus* was isolated did not fulfil criteria for allergic bronchopulmonary aspergillosis and no patients were on treatment with oral steroids [26]. The 2 patients from whom nontuberculous mycobacteria were isolated did not fulfil criteria for nontuberculous mycobacterial lung disease [27].

### Primary endpoint: MRSA eradication

The per-protocol analysis indicated that MRSA was eradicated in 12 (63.2%) out of 19 patients in arm A while spontaneous clearance was observed in 5 (38.5%) out of 13 patients in arm B. There was a 24.7% (z = 1.37, P(Z>z) = 0.08, one-tailed) difference in MRSA clearance between the two groups.

In arm A 11 (91.7%) out of 12 patients in whom MRSA was eradicated had a first infection and only 1 patient was previously infected.

Two patients in arm A stopped treatment due to untoward effects which were probably attributable to the therapy (1 patient with vomiting and another with urticaria), although the severity of the effects did not require hospitalization.

### Secondary endpoints

#### 1. FEV_1_ and BMI change

At 6 months following the treatment period, the mean (±SD) absolute change in FEV_1_ (percentage of predicted) from baseline was 7.13% (±14.92) in 15 patients able to perform spirometry in arm A and -1.16% (±5.25) in 12 patients able to perform spirometry in arm B (p = 0.08). In the same period the BMI change (mean ±SD) from baseline was 0.54 (±1.33) kg/m^2^ in 19 patients in arm A and -0.38 (±1.56) kg/m^2^ in 13 patients in arm B (p = 0.08).

#### 2. MRSA*-*free period

Table 2 illustrates the percentage of MRSA-free patients for each of the three cultures in both arms of the study over 6 months. The limited number of patients in the study entails a degree of uncertainty regarding this estimation with overlapping confidence intervals.

**Table 2:** MRSA-free patients at 1^st^, 2^nd^, and 3^rd^ culture for treatment and observational arm. Percentage of MRSA-free patients and 95% CI calculated using exact likelihood.

	Enrolled Patients	MRSA*-*free Patients	Patients with MRSA isolation	Percentage of MRSA-free patients
	n	n	n	(95% CI)
**Arm A** (29 patients)				
Drop-out	10			
Completed follow-up	19			
at 1^st^ culture (60 days)		14	5	74 (49–91)
at 2^nd^ culture (120 days)		13	6	68 (43–87)
at 3^rd^ culture (180 days)		12	7	63 (38–84)
**Arm B** (32 patients)				
Drop-out	19			
Completed follow-up	13			
at 1^st^ culture (60 days)		7	6	54 (25–81)
at 2^nd^ culture (120 days)		6	7	46 (19–75)
at 3^rd^ culture (180 days)		5	8	38 (14–68)

#### 3. Microbiological status of patients at six months

As shown in Table 3, after assessment of the microbiological status of patients at 6 months, we observed an increase in patients infected by *S*. *aureus* (MSSA) in both arms of the study. No substantial change in the prevalence of *P*. *aeruginosa*, *A*. *xylosoxidans* and *S*. *maltophilia* infections was observed. No *B*. *cepacia* complex was isolated from patients in either arm of the study.

**Table 3:** Co-infection with other pathogens at 6 months.

	Arm A	Arm B
Number of patients	n = 19	n = 13
***S*. *aureus* (MSSA)**		
Positive	11 (57.9%)	8 (61.5%)
Negative	8 (42.1%)	5 (38.5%)
***P*. *aeruginosa***		
Positive [chronic]	4 (21.1%) [4]	5 (38.5%) [4]
Negative	15 (78.9%)	8 (61.5%)
***A*. *xylosoxidans***		
Positive	1 (5.3%)	0 (0%)
Negative	18 (94.7%)	13 (100%)
***S*. *maltophilia***		
Positive	1 (5.3%)	4 (30.8%)
Negative	18 (94.7%)	9 (69.2%)
***Aspergillus* spp**		
Positive	0 (0%)	3 (23.1%)
Negative	19 (100%)	10 (76.9%)
**Other**		
Positive	5 (26.3%) 2 with *B*. *gladioli* 3 with *H*. *influenzae*	2 (15.4%) 1 with nontuberculous mycobacterium 1 with *H*. *influenzae*

#### 4. Pulmonary exacerbations and use of antibiotics

During the time of the study 6 (31.6%) out of 19 patients in arm A and 4 (30.8%) out of 13 patients in arm B were treated with intravenous antibiotics. No statistically significant difference was observed in the number of patients treated intravenously between the 2 arms of the study (p = 1). Pulmonary exacerbations [17], hospitalizations and days of total (oral, inhaled and intravenous) antibiotic usage were assessed in the active and observational arms for 6 months (Table 4). We found no statistically significant differences between patients in arm A and arm B.

**Table 4:** Pulmonary exacerbations, days spent in hospital and antibiotic use over 6 months of study.

	Arm A (19 patients)	Arm B (13 patients)	p value	Difference (95% CI)
Exacerbations (number)	1.00±0.82	0.85±1.07	0.66	0.15 (-0.53 to 0.83)
Days spent in hospital (mean±SD)	4.26±6.16	3.62±6.56	0.78	0.64 (-4.01 to 5.29)
Intravenous treatment (days) (mean±SD)	6.37±9.77	6.38±10.67	0.99	0.01 (-7.46 to 7.44)
Oral treatment^a^ (days) (mean±SD)	39.47±32.95	41.08±45.84	0.91	-1.61 (-30.00 to 26.78)
Inhalation treatment (days) (mean±SD)	44.47±64.12	47.77±43.37	0.87	-3.33 (-45.01 to 38.41)

^a^ including azithromycin treatment

#### 5. Microbiological data

The susceptibility pattern of all MRSA strains at baseline is shown in Table 5. Molecular analysis was performed on the 18 (29.5%) out of 61 MRSA isolates which arrived at the Central laboratory. The distribution of SCC*mec* types, PVL production, *spa* types and ST among patients are described in Table 6. The most frequent clones were ST22-IV (27.8%) and ST1-IV (16.6%) and the most represented SCC*mec* types were type IV (67%), followed by types V and I (11%) and type III (5%) [20,21]. Only 2 isolates were positive for the PVL gene.

**Table 5:** Percentage of susceptibility of 61 MRSA strains at baseline.

Antimicrobial	%		
	Sensitive	Intermediate	Resistant
Clindamycin ^1^	38	0	62
Daptomycin	98	0	2
Tigecycline	98	0	2
TMP/SMX	98	0	2
Linezolid	93.9	0	6.1
Rifampicin	92	0	8
Mupirocin	92	6	2
Fusidic acid	86.3	0	13.7
Moxifloxacin	64.3	28.6	7.1
Teicoplanin	100	0	0
Vancomycin	100	0	0
Tetracycline	64	4	32
Gentamicin	61.8	2.9	35.3
Levofloxacin	56.9	1.9	41.2

^1^ Clindamycin is reported as total resistance (constitutive plus inducible).

**Table 6:** The distribution of SCC*mec* types, PVL status and Sequence Types (ST) on 18 (29.5%) out of 61 MRSA isolates.

Clone name	ST	SCC*mec*	*spa* type	PVL	Patients N.
ST1-IV	1	IV	t127/n.id.	negative	3
ST1-V	1	V	t127	negative	1
ST5-III	5	III	t002	negative	1
ST8-IV	8	IV	t008	negative	1
ST15-I	15	I	n.id.	negative	1
ST22-IV	22	IV	t852/t1977	1 positive	5
ST59-V	59	V	t216	negative	1
ST97-IV	97	IV	t359	negative	1
ST398	398	n.id.	t108	negative	1
n.id.	n.id.	I (1)/IV(2)	t019/n.id.	1 positive	3

## Discussion

The present study was designed to evaluate the efficacy of an early eradication treatment protocol lasting 21 days against new-onset MRSA infection in CF patients in various centers around Italy, where the prevalence of this pathogen is only partially known [1, 25]. Over a 6- month period, we observed a 24.7% difference in MRSA clearance between patients in the active arm A and observational arm B of the study, according to per-protocol statistical analysis. During the same period, we also saw positive effects in respiratory function (FEV_1_), nutritional status (BMI) and MRSA-free period in the patients of the active arm. The microbiological analysis of patients provided indications of sensitivity to the antibiotics and molecular characteristics of a subset of MRSA strains responsible for the new-onset infection.

The principal limitations of the present study are the limited statistical power, the high drop-out number and the per-protocol analysis, that could overestimate the effectiveness of the intervention [18]. Despite these limits, our study helps to gain experience in the treatment of new-onset MRSA infection in CF and gives credence to the idea that early intervention could increase the clearance of this pathogen. The slow recruitment of patients to this study was due to overestimation of the incidence of MRSA infection in Italy, while designing the trial [25]. The epidemiology of this infection is not currently defined by the European CF Society Registry [1] and can vary notably from place to place.

Antibiotics potentially active against MRSA are sometimes used in CF patients co-infected with other pathogens. In both study arms the high drop-out percentage can be attributed to the need to administer additional antibiotics against MRSA due to worsening clinical conditions or to the use of other antibiotics to treat *P*. *aeruginosa* infection [28]. Although tobramycin and quinolones are not usually prescribed to treat MRSA infections [24,29], we consider the use of such drugs as a reason for drop-out given that their activity against MRSA cannot be excluded [24, 25, 30, 31].

The difference in MRSA clearance between the 2 arms of the study confirms previous observations [5–10] regarding the microbiological efficacy of the treatment and reinforces the idea of eradicating this pathogen during its initial phases of infection. It can be hypothesized that early treatment can clear the pathogen before it has a chance to adapt to the respiratory tract of the CF patient, as seen with *P*. *aeruginosa* [32, 33].

In our study, treatment was started quickly and the average time between new-onset MRSA infection diagnosis and the time of treatment initiation was in accordance with the times in the best practice guidelines against *P*. *aeruginosa* [15]. Moreover, the measure outcome was based on three microbiological cultures carried out over a period of 6 months, thereby complying with the definitions normally used in clinical practice to describe the efficacy of eradicating treatment [11,13, 24]. The definition of eradication used in other experiences, based on a single culture or on limited observation period fails to satisfy the definition of eradication normally used for other pathogens [5, 6, 8, 10].

The phenomenon of spontaneous clearance of the pathogen has been described for some time, and we observed a proportion in our observational arm analogous to that found in other studies [5–10]. The explanation for this phenomenon is currently unknown.

Although we were unable to enroll as many participating patients as we intended, our results should be evaluated from a clinical point of view. A difference of 24.7% in pathogen clearance between the active and the observational arm is an important difference.

The possibility of eradicating MRSA in the initial phases of infection, [5–10], the considerable worsening of clinical conditions due to persistent MRSA infection [3,4] and other observations, such as the consistently unfavorable effect on the course of the disease when the patient has MRSA and *P*. *aeruginosa* co-infection [34,35], should reinforce the decision to intervene rapidly. Our experience, together with results from previous studies, could contribute to proposals which include early MRSA eradication treatment as part of the standard of care of CF patients.

Our results can be generalized clinically. We respected the definitions of eradication which are also valid for other pathogens and considered proper clinical practice [11–13]. We enrolled a group of patients with variable characteristics of age, sex and disease severity. The mean age of our patients was analogous to the age of other subjects infected by MRSA [2]. We cannot exclude the possibility that patients even younger than ours are infected by MRSA strains, but in these cases, treatment efficacy remains to be demonstrated.

Our microbiological analysis performed on a subset of strains responsible for the infection in our study population indicates that they were mainly community-acquired MRSA (CA-MRSA). This observation brings up the problem of environmental and skin decontamination [11]. Cutaneous and/or environmental decontamination might be somewhat difficult [10,11,36–38] as patients may not easily accept this practice on a daily basis. Our protocol, involving use of only one hygienic measure aimed at reducing nasal colonization, (rather than cutaneous and environmental decontamination), was probably easier for the patient to accept and simpler to carry out than other previous experiences [10]. The assessment of the efficacy of the different practices of environmental, nasal and skin decontamination in reducing over time the phenomenon of MRSA pulmonary re-colonization needs further studies.

The results from our study seem to indicate that the risk of acquiring an infection due to other non-fermenter Gram negatives pathogens was not consistent. During the study, in both arms, we observed an increase in MSSA co-infection at 6 months. The reasons and the clinical significance of this phenomenon are not known. The strategies adopted by the Centers participating in the study regarding the MSSA infection were not investigated. Unlike *P*. *aeruginosa* strategies, where there is universal consensus regarding the approach [15], there is no agreement regarding MSSA infection [39]. Furthermore, the follow-up period of the present study did not allow us to ascertain whether the MSSA increase was transitory or persistent. [1,15].

The eradication protocol that we chose to evaluate in this trial is based on patterns of sensitivity of the MRSA isolates in Italy [25], on the cost of the pharmaceuticals used, and the fact that some drugs which are active against MRSA, such as fusidic acid, are not currently available in Italy. The drugs which should be used in an early-eradication protocol of MRSA obviously need to be selected on the basis of antibiotic-susceptibility of the strains responsible for the new-onset infection, which are often resistant to various drugs [24,29, 40]. This fact means that it is not possible to recommend a gold standard since physicians must often select alternative protocols based on the resistance patterns of the local strains affecting their patients. Due to the limited experience in the field of MRSA eradication in CF, other studies are definitively necessary. Large-scale studies designed to compare the clinical efficacy of various types of treatments are theoretically feasible [41] but the growing number of patients involved in clinical trials may make it difficult to recruit the number of subjects necessary to reach statistical power. Moreover, running clinical trials on MRSA new infection is made more difficult because of absence of data on MRSA prevalence in certain countries, the need to treat patients with antibiotics potentially active against MRSA and the lack of a universally accepted definition of eradication.

In our study, the antibiotics have been well tested in the clinic and do not pose a significant risk of side effects. Although individual allergic reactions in patients cannot be excluded, the classes of antibiotics which are effective against MRSA are less allergenic than other classes (such as the beta-lactams) [42]. We cannot exclude the possibility that antibiotics by aerosol inhalation are used in the early phases of the infection. Clinical trials on the use of vancomycin against persistent MRSA infection are ongoing [43].

In conclusion, the results of our study agree with previous experiences regarding the possibility of eradicating new-onset MRSA infection [5–10], and show favorable effects in CF patients’ FEV_1_ and BMI over a period of 6 months. These results, together with other data from the literature and the low risk of side effects of the treatment, suggest that this strategy could be more widely implemented in the treatment of CF patients.

## Supporting information

S1 File CONSORT checklist. (PDF)

S2 File Trial study protocol. (PDF)

S3 File Trial study protocol (original language). (PDF)

S1 Dataset Primary outcome data. (XLS)

S2 Dataset Arm A. Days in hospital and treatments.
(XLS)

S3 Dataset Arm B. Days in hospital and treatments.
(XLS)

S4 Dataset Variation BMI at 6 months in both arms. (XLSX)

S5 Dataset Variation in FEV1 at 6 months in both arms. (XLSX)

## Abbreviations

MRSA: Methicillin-resistant *Staphylococcus aureus*
FEV_1_: Forced Expiratory Volume in the 1st second
BMI: Body Mass Index
ST: Sequence Type
PVL: Panton-Valentine Leukocidin
SCC*mec*: staphylococcal cassette chromosome mec
