# Supplementary material for: Methicillin-resistant Staphylococcus aureus eradication in cystic fibrosis patients: A randomized multicenter study
Source: PLoS One. 2019 Mar 22;14(3):e0213497. doi: 10.1371/journal.pone.0213497 (PMC6430412; doi:10.1371/journal.pone.0213497)
Supplement: S2 File — (PDF) [file pone.0213497.s002.pdf]

Early eradication of methicillin-resistant *Staphylococcus aureus* infection in cystic fibrosis patients: a randomized multi center trial

(Version October 1st, 2012)

Rationale

The incidence of methicillin-resistant *Staphylococcus aureus* (MRSA) infections is increasing in cystic fibrosis (CF) patients and is associated with a consistent decline in their FEV_1 rates and increased mortality. Although it is possible for MRSA to clear spontaneously from the respiratory tract of CF patients, early eradication treatment can increase the percentage of eradication of the bacteria, thus guaranteeing that a higher number of patients experience a relevant MRSA-free period. Early eradication followed by a period when the patient is clinically MRSA-free is particularly important because persistence of this bacteria is a negative prognostic factor for CF patients.

There are very few studies available which have analyzed the possibility of early eradication of MRSA. There are currently no published data on large numbers of patients which compare the efficacy of early eradication treatment with observation alone.

Study design

Prospective randomized controlled study

Objective

The primary aim of this randomized, multicenter study is to compare, using statistical methods, the efficacy of an early eradication treatment of a first or new MRSA infection, with oral cotrimoxazole and rifampicin in association with nasal mupirocin, in a consistently ample group of CF patients. There will be an active arm of the study (A) versus an observation-only arm (B).

Glossary:

- First MRSA infection: First isolation of the germ from the airways of CF patients.
- New MRSA infection: Each successive isolation of the germ after a clearance period of 12 months (after 4 negative cultures).
- Eradication of MRSA: Absence of the germ following antibiotic treatment in at least 3 cultures performed over a period of 6 months.

The secondary aims of this study are:

1. to assess the change in forced expiratory volume in one second (FEV_1) and body mass index (BMI) of patients during a time span of 6 months;
2. to determine the existence of any differences between the two arms in regard to the period in which the patient remains MRSA-free;
3. to determine if the eradication treatment is associated with an increasing risk of emergence of other pathogens (*Burkholderia cepacia* complex and other non-fermentative Gram-negative bacteria) in the patient's respiratory tract;
4. to assess the number of pulmonary exacerbations and hospitalizations, the days of total (oral, inhaled and intravenous) antibiotic usage, in the two arms over a period of 6 months;
5. to evaluate the antibiotic susceptibility and molecular characteristics of MRSA strains isolated from the airways of CF patients experiencing initial MRSA infection.

Study population

<u>Participants:</u>

Inclusion criteria

Patients in regular (quarterly) clinical and microbiological follow-up will be considered eligible if they are more than 4 years old and experiencing an initial MRSA infection.

Exclusion criteria

Patients will be excluded from the study in case of:

- Respiratory exacerbation (Fuchs HJ et, 1994) at the time of randomization
- History of hypersensitivity to or adverse reaction to antibiotics
- Liver cirrhosis or abnormal liver function test results at study entry (defined as ALT and/or AST levels more than twice the upper limit of the normal range)
- Abnormal kidney function at study entry (defined as a serum creatinine level >1.5 times the upper limit normal for the participant's age)
- Pregnancy
- Lung/liver transplantation
- *Burkholderia cepacia* complex infection

- MRSA resistance to both antibiotics, trimethoprim/sulfamethoxazole (TMP/SMX) and rifampicin
- Contemporaneous use of any investigational drug

All patients will give informed consent for inclusion in the study. Demographic and baseline characteristics will be summarized.

Randomization

CF patients with a first/new MRSA infection will be randomly assigned to the active arm (A) or observational arm (B). A balanced randomization sequence with permuted blocks of size 4 will be created using statistical software. Randomization assignment, performed at the coordinator Center (Meyer Hospital), will be organized by e-mail.

Patients will be allocated 1:1 and distributed into two groups, stratified according to FEV_1 values (≥70% or <70%). Participants will be enrolled at their own CF Center. The people involved in randomization and in the treatment assignments will be kept completely separate.

Swabs and sputum collection

Oropharyngeal swabs or sputum will be collected at every visit for microbiological analyses. Swabs will be taken on both tonsillar areas and the posterior pharynx. Sputum will be collected in a sterile tube.

Treatment:

Patients randomized to the active arm (A) will be treated with the following antibiotic regimen:

- Oral rifampicin 15 mg/kg/day in 2 daily doses (maximum daily dose 600 mg) for 21 days
- Oral TMP-SMX 8-40 mg/kg/day in 2 daily doses (maximum daily dose 320/1600 mg) for 21 days
- 2% nasal mupirocin – each nostril 3 times daily for 5 days

In case of antibiotic resistance, an alternative approach has been planned: patients over 8 years of age will be treated with rifampicin and minocycline when MRSA is resistant to TMP/SMX, or with TMP/SMX and minocycline (pediatric dose: 2 mg/kg orally twice daily for 21 days, adult dose: 100 mg orally twice daily for 21 days) when MRSA is resistant to rifampicin.

Treatment will be suspended in cases of adverse effects

The decision of how to medically proceed in cases of persistence of colonization notwithstanding treatment or in cases of respiratory exacerbation (Fuchs HJ et al 1994) during treatment will be left to the discretion of the individual Center.

Outcome measures

The primary outcome will be MRSA eradication, defined as the patient having three successive negative cultures in 6 months according to the United Kingdom CF Trust criteria. The difference in clinical interest between the experimental treatment (arm A) and observation is 25%.

Having received antibiotics active against MRSA during the follow-up will be considered a cause of drop-out.

Sample size and power

This trial is designed by calculating the sample size as a balance between statistical considerations and epidemiological experience of MRSA infection in Italy (Cocchi P et al. 2011). Data regarding MRSA infection are not available in the Italian CF Registry. Approximately 35 patients per year will be enrolled in the study.

The null hypothesis will be that there is no difference between the treatment group and the observational group. The alternative hypothesis will be that there is a difference when the experimental treatment is better than the observation group (control). A 25% greater rate of eradication in arm A in comparison with arm B will be considered clinically relevant. Data will be independent, with one observation per participant. The error probability of the first type α will be set at 0.05.

By hypothesizing that MRSA eradication will occur in 80% of cases in the treated group (arm A) and that spontaneous clearance will occur in 50% of the untreated group (arm B), it will be necessary to enroll 100 patients (50 per arm) to reach statistical significance, considering that a 25% difference between the two arms of the study is clinically relevant.

Two Independent Proportions (Null Case) Power Analysis

Page/Date/Time 1 24/01/2012 11.07.14

Numeric Results of Tests Based on the Difference: P1 - P2

H0: P1-P2=0. H1: P1-P2=D1<>0. Test Statistic: Z test with unpooled variance

Power	Sample Size Grp 1 N1	Sample Size Grp 2 N2	Prop|H1 Grp 1 or Trtmnt P1	Prop Grp 2 or Control P2	Diff if H0 D0	Diff if H1 D1	Target Alpha	Actual Alpha	Beta
0.3188	10	10	0.8000	0.5500	0.0000	0.2500	0.0500	0.0895	0.6812
0.3623	15	15	0.8000	0.5500	0.0000	0.2500	0.0500	0.0589	0.6377
0.4562	20	20	0.8000	0.5500	0.0000	0.2500	0.0500	0.0794	0.5438
0.5200	25	25	0.8000	0.5500	0.0000	0.2500	0.0500	0.0642	0.4800
0.5742	30	30	0.8000	0.5500	0.0000	0.2500	0.0500	0.0518	0.4258
0.6444	35	35	0.8000	0.5500	0.0000	0.2500	0.0500	0.0708	0.3556
0.6880	40	40	0.8000	0.5500	0.0000	0.2500	0.0500	0.0556	0.3120
0.7466	45	45	0.8000	0.5500	0.0000	0.2500	0.0500	0.0516	0.2534
0.7842	50	50	0.8000	0.5500	0.0000	0.2500	0.0500	0.0557	0.2158

Note: exact results based on the binomial were only made when both N1 and N2 were less than 100.

Report Definitions

'Power' is the probability of rejecting a false null hypothesis. It should be close to one. 'N1 and N2' are the sizes of the samples drawn from the corresponding populations.

'P1' is the proportion for group one under H1. This is the treatment or experimental group. 'P2' is the proportion for group two. This is the standard, reference, or control group 'Target Alpha' is the probability of rejecting a true null hypothesis that was desired. 'Actual Alpha' is the value of alpha that is actually achieved.
'Beta' is the probability of accepting a false null hypothesis.

Summary Statements

Group sample sizes of 10 in group one and 10 in group two achieve 32% power to detect a difference between the group proportions of 0.2500. The proportion in group one (the treatment group) is assumed to be 0.5500 under the null hypothesis and 0.8000 under the alternative hypothesis. The proportion in group two (the control group) is 0.5500. The test statistic used is the two-sided Z test with unpooled variance. The significance level of the test was targeted at 0.0500. The significance level actually achieved by this design is 0.0895.

<u>Methods used to evaluate primary outcome and compare results obtained in the groups</u>

Data will be analyzed involving all patients under study. Eradication is defined as three successive negative cultures in 6 months according to the United Kingdom CF Trust criteria. Difference in MRSA clearance between the active and the observational groups will be calculated. The results will be presented in absolute numbers and overall results (OR) with a 95% confidence interval.

<u>Other procedures to evaluate secondary aims</u>

Results of cultures and clinical records will be used to assess secondary aims. Patients' microbiological status will be determined according to the European CF Registry definitions.

FEV_1 values will be measured according to ATS-ERS standards.

<u>Microbiology</u>

Samples obtained from the airways (sputum or oropharyngeal swabs) will be analyzed. All the samples will be processed for detection of methicillin-resistant *Staphylococcus aureus* (MRSA) isolates using selective culture media. After 48 hours of incubation in an aerobic atmosphere at 37°C, the plates will be read to detect the presence of pathogens. The isolation of *S. aureus* bacteria will be based on typical colony morphology using selective culture media (Mannitol Salt Agar 2, bioMérieux) and chromogenic culture media for MRSA (BBL™ CHROMagar™ MRSA II, Becton Dickinson). In order to confirm species-level identification, biochemical tests will be performed using Vitek2 (bioMérieux).

The antibiotic susceptibility profiles will be evaluated using the VITEK®2 (bioMérieux) automated system and EUCAST clinical breakpoints will be used as interpretation criteria [www.eucast.org/clinical_breakpoints/]. All identified strains will be stored at -80°C for subsequent molecular analysis.

DNA extraction will be performed from pure *S. aureus* cultures after 24 hours of incubation at 37°C on Columbia agar + 5% sheep's blood (bioMérieux) using QIAamp DNA Mini Kit (cat. num. 51306, QIAGEN, Netherlands) according to the manufacturer's specifications

To determine the potential virulence of MRSA strains, a specific PCR assay for the presence of the Panton-Valentine Leukocidin (PVL) gene will be performed [Lina et al, 1999].

During the study period, the *mecA* gene and other loci of the SCC*mec* cassette will be characterized following different multiplex PCR. The protocol suggested by Oliveira and de Lancastre in 2002 will be used as a first screening test to be confirmed by the method published by Zhang and colleagues in 2005. New methods have been recently developed to highlight the presence of SCC*mec* types VI, VII and VIII (Mileiriço et al, 2006; Higuchiet al, 2008; Zhang K et al, 2009, b), and will be applied to highlight the presence of these particular SCC*mec* types. MLST analyses will be based on the nucleotide sequences of 7 different housekeeping genes: isolates will be defined by the alleles present at the seven loci (the allelic profile), and each unique allelic profile will be assigned a sequence type (ST). Isolates with the same ST will therefore have identical sequences at all seven MLST loci and will be considered to be members of a single clone (Enright et al, 2000).

<u>Statistical methods.</u>

During the same 6-month period, we will assess the patients' FEV_1 change (% of predicted), nutritional status (BMI), pulmonary exacerbations (number), days spent in hospital and antibiotic use (days in treatment) in the active (A) and in the observational (B) arm.

Secondary aims will be evaluated using descriptive statistics, parametric and nonparametric statistical procedures on the differences in continuous and categorical variables between groups and

comparisons of estimated MRSA-free period. The level of significance will be set at 5%. Results for quantitative variables will be expressed as mean ±SD.
