# Supplementary material for: Methicillin-resistant Staphylococcus aureus eradication in cystic fibrosis patients: A randomized multicenter study
Source: PLoS One. 2019 Mar 22;14(3):e0213497. doi: 10.1371/journal.pone.0213497 (PMC6430412; doi:10.1371/journal.pone.0213497)
Supplement: S3 File — (PDF) [file pone.0213497.s003.pdf]

Eradicazione precoce delle infezioni da *Staphylococcus aureus* meticillino resistente nei pazienti affetti da fibrosi cistica: uno studio randomizzato multicentrico
(Versione 1 ottobre 2012)

Razionale

La prevalenza delle infezioni da *Staphylococus aureus* meticillino-resistente (MRSA) è in aumento nei pazienti affetti da fibrosi cistica (FC) ed è stata associata a un parallelo declino del FEV_1 e ad un incremento della mortalità. Nonostante sia segnalata la possibilità di una clearance spontanea di MRSA dalle vie aeree dei pazienti affetti da FC, il trattamento antibiotico eradicante precoce potrebbe incrementare la possibilità di clearance del batterio e garantire così un tempo libero dall'infezione a un numero maggiore di pazienti. L'eradicazione precoce di MRSA e il conseguente tempo libero dall'infezione appaiono di particolare rilevanza clinica, in quanto la persistenza di MRSA a livello delle vie aeree è oggi considerata un fattore prognostico negativo per i pazienti affetti da FC. Tuttavia finora pochi studi hanno indagato la possibilità di eradicazione precoce di MRSA, ed in particolare non esistono allo stato attuale studi su larga scala che comparino l'efficacia del trattamento eradicante rispetto alla semplice osservazione.

Disegno dello studio

Studio prospettico randomizzato controllato multicentrico.

Obiettivo

L'obiettivo primario dello studio è valutare con metodi statistici l'efficacia di un trattamento antibiotico, con cotrimossazolo e rifampicina *per os* in associazione all'applicazione nasale di mupirocina, nell'eradicazione di una prima o recente infezione da MRSA in un gruppo di pazienti affetti da FC. Lo studio prevederà un braccio attivo (A) e un braccio di controllo di semplice osservazione (B).

Glossario

- Prima infezione da MRSA: primo isolamento del batterio dalle vie aeree di un paziente.
- Recente infezione da MRSA: ogni isolamento del batterio successivo al primo, dopo un periodo di clearance di 12 mesi documentato da almeno 4 colture negative.
- Eradicazione di MRSA: assenza del batterio in almeno 3 colture eseguite nell'arco di almeno 6 mesi dopo il trattamento antibiotico.

Obiettivi secondari dello studio sono:

1. valutazione del cambiamento di FEV_1 e dell'indice di massa corporea (IMC) nei pazienti in un arco temporale di 6 mesi;
2. valutazione di possibili differenze nel periodo libero dall'infezione fra i due bracci;
3. valutazione dell'eventuale aumento del rischio di emergenza di nuovi patogeni nelle vie aeree dei pazienti dopo il trattamento antibiotico eradicante (*Burkholderia cepacia* complex ed altri batteri Gram-negativi non fermentanti);
4. valutazione del numero di riacutizzazioni polmonari, della necessità di ospedalizzazione, nonché dei giorni di terapia antibiotica (orale, inalatoria e paranterale) nei due bracci in un arco temporale di 6 mesi;
5. valutazione della suscettibilità agli antibiotici e delle caratteristiche molecolari dei ceppi di MRSA isolati dalle vie aeree dei pazienti affetti da FC in corso di infezione nuova o recente.

Popolazione in studio

Partecipanti

Criteri di inclusione

Pazienti affetti da FC con infezione recente da MRSA ed età maggiore a 4 anni, che eseguono controlli clinici e microbiologici a cadenza regolare, ossia ogni 3 mesi.

Criteri di esclusione

Saranno esclusi dallo studio i pazienti che presentassero:

- Riacutizzazione respiratoria (Fuchs HJ et, 1994) al momento della randomizzazione;
- Storia personale di ipersensibilità o pregresse reazioni avverse agli antibiotici;
- Cirrosi epatica o alterazioni della funzionalità epatica al momento dell'arruolamento (definite come elevazione di ALT e/o AST oltre due volte il limite superiore di norma);
- Alterazioni della funzionalità renale al momento dell'arruolamento (definito come aumento dei livelli di creatinina sierica > 1,5 volte il limite superiore di norma per età);
- Gravidanza;
- Trapianto di fegato e/o polmone;
- Infezione da *Burkholderia cepacia* complex;
- Infezione da MRSA resistente a entrambi gli antibiotici, TMP/SMX e a rifampicina;
- Uso concomitante di qualunque altro farmaco in regime sperimentale.

Tutti i pazienti sottoscriveranno un consenso informato al momento dell'arruolamento. Saranno descritte le caratteristiche demografiche dei pazienti inclusi in ognun braccio di trattamento.

Randomizzazione

I pazienti inclusi nello studio saranno assegnati in maniera casuale al braccio attivo (A) o al braccio osservazionale (B): tramite uno specifico software statistico verrà generata una sequenza di randomizzazione bilanciata in blocchi permutati di 4. I pazienti saranno allocati 1:1 e distribuiti nei 2 gruppi stratificati in base al relativo FEV1 (≥70% o <70%). I pazienti saranno arruolati nel centro di FC presso cui sono seguiti. Gli sperimentatori coinvolti nella randomizzazione e nell'allocazione saranno separati dai medici coinvolti nel trattamento dei pazienti. La randomizzazione e l'assegnazione a uno dei due bracci avverranno via e-mail.

Raccolta dei campioni per studio microbiologico

Ad ogni valutazione clinica saranno eseguiti tamponi orofaringei o raccolta dell'espettorato dei pazienti per eseguire le analisi microbiologiche.
I tamponi orofaringei saranno effettuati toccando con il tampone entrambe le tonsille e a livello della faringe posteriore. L'espettorato sarà raccolto in contenitori sterili.

Schema di trattamento

Lo schema di trattamento riservato ai pazienti assegnati al braccio attivo (A) è il seguente:

- rifampicina orale 15 mg/kg/die in 2 dosi giornaliere (fino a un massimo dosaggio giornaliero di 600 mg) per 21 giorni;
- TMP-SMX orale 8-40 mg/kg/die in 2 dosi giornaliere (fino a un massimo dosaggio giornaliero di 320/1600 mg) per 21 giorni;
- mupirocina nasale 2%: applicazione topica in ogni narice 3 volte/die per 5 giorni.

Nel caso in cui il batterio mostrasse resistenza a rifampicina o a TMP/SMX è previsto il seguente approccio alternativo: i pazienti di età superiore agli 8 anni riceveranno rifampicina e minociclina qualora MRSA fosse resistente a TMP/SMX, mentre riceveranno TMP/SMX e minociclina qualora MRSA fosse resistente a rifampicina. La minociclina sarà somministrata per os al dosaggio pediatrico (2 mg/kg/die suddivisi in due dosi giornaliere per 21 giorni). La dose per adulti sarà somministrata ad un dosaggio di 100 mg 2 volte al dì per 21 giorni.
Il trattamento sarà sospeso nel caso si verificassero effetti avversi.

In caso di persistenza del germe nelle vie aeree (fallimento di un singolo ciclo di eradicazione) o in caso di esacerbazione respiratoria (Fuchs HJ et al NEJM 1994), la decisione terapeutica verrà lasciata all'esperienza dei medici del centro di riferimento del paziente.

Misure di outcome

L'outcome primario sarà l'eradicazione di MRSA, definita come negatività di MRSA in almeno 3 colture da tampone faringeo e/o espettorato nell'arco di 6 mesi, secondo i criteri della CF Trust del Regno Unito (UK). La differenza di interesse clinico fra il braccio sperimentale (A) e il braccio osservazionale (B) è posta al 25%. Saranno esclusi dallo studio i pazienti che riceveranno antibiotici attivi nei confronti di MRSA durante il periodo di follow-up.

Dimensioni del campione e potenza dello studio

Nel progetto di studio la dimensione del campione è stata calcolata combinando considerazioni statistiche e dati epidemiologici relativi all'infezione da MRSA in Italia (Cocchi P et al., 2011). Allo stato attuale i dati relativi all'infezione da MRSA non sono disponibili nel Registro Italiano dei pazienti affetti da FC. È previsto un arruolamento di circa 35 pazienti all'anno.

L'ipotesi nulla sarà che non ci sia differenza tra il braccio attivo (A) e il braccio di osservazione (B). L'ipotesi alternativa sarà che esiste una differenza e che il trattamento sperimentale sia migliore dell'osservazione. Sarà considerato clinicamente rilevante un tasso di eradicazione maggiore del 25% nel braccio A rispetto al braccio B. I dati saranno indipendenti, con un'osservazione per partecipante. La probabilità di errore del primo tipo α sarà impostata a 0,05.

Ipotizzando che l'eradicazione di MRSA si verifichi nell'80% dei casi nel gruppo trattato (braccio A) e che la clearance spontanea si verifichi nel 50% del gruppo non trattato (braccio B), sarà necessario arruolare almeno 100 pazienti (50 per braccio) per raggiungere la significatività statistica, dal momento che si considera clinicamente rilevante una differenza del 25% tra i due bracci dello studio.

Lo studio di potenza è illustrato nella seguente tabella:

Two Independent Proportions (Null Case) Power Analysis

Page/Date/Time 1 24/01/2012 11.07.14

Numeric Results of Tests Based on the Difference: P1 - P2
H0: P1-P2=0. H1: P1-P2=D1<>0. Test Statistic: Z test with unpooled variance

Power	Sample Size Grp 1 N1	Sample Size Grp 2 N2	Prop|H1 Grp 1 or Trtmnt P1	Prop Grp 2 or Control P2	Diff if H0 D0	Diff if H1 D1	Target Alpha	Actual Alpha	Beta
0.3188	10	10	0.8000	0.5500	0.0000	0.2500	0.0500	0.0895	0.6812

0.3623	15	15	0.8000	0.5500	0.0000	0.2500	0.0500	0.0589	0.6377
0.4562	20	20	0.8000	0.5500	0.0000	0.2500	0.0500	0.0794	0.5438
0.5200	25	25	0.8000	0.5500	0.0000	0.2500	0.0500	0.0642	0.4800
0.5742	30	30	0.8000	0.5500	0.0000	0.2500	0.0500	0.0518	0.4258
0.6444	35	35	0.8000	0.5500	0.0000	0.2500	0.0500	0.0708	0.3556
0.6880	40	40	0.8000	0.5500	0.0000	0.2500	0.0500	0.0556	0.3120
0.7466	45	45	0.8000	0.5500	0.0000	0.2500	0.0500	0.0516	0.2534
0.7842	50	50	0.8000	0.5500	0.0000	0.2500	0.0500	0.0557	0.2158

Metodi impiegati per valutare l’outcome primario e per confrontare i risultati ottenuti nei gruppi:

I dati saranno analizzati coinvolgendo tutti i pazienti arruolati. L'eradicazione è definita come tre colture negative successive nell’arco di almeno 6 mesi secondo i criteri UK CF Trust. Sarà calcolata la differenza di clearance dell'MRSA fra il braccio attivo (A) e il braccio osservazionale (B). I risultati saranno presentati in numeri assoluti e i risultati complessivi come OR con un intervallo di confidenza del 95%.

Altre procedure per valutare gli obiettivi secondari

Gli obiettivi secondari saranno valutati ricorrendo ai dati contenuti nella documentazione clinica e ai risultati delle colture. Lo stato microbiologico dei pazienti sarà determinato in base alle definizioni del Registro Europeo FC.

I valori FEV_1 saranno misurati in base agli standard ATS-ERS.

Microbiologia

Verranno analizzati i campioni ottenuti dalle vie aeree (espettorato o tamponi orofaringei). Tutti i campioni saranno processati per il rilevamento di ceppi di *Staphylococcus aureus* meticillino resistenti (MRSA) usando terreni di coltura selettivi. Dopo 48 ore di incubazione in atmosfera aerobica a 37 ° C, le piastre saranno lette per rilevare la presenza di colonie batteriche. L'isolamento di *S. aureus* sarà basato sulla tipica morfologia delle colonie utilizzando terreni di coltura selettivi (Mannitol Salt Agar 2, bioMérieux) e terreni di coltura cromogenici per MRSA (BBL ™ CHROMagar ™ MRSA II, Becton Dickinson). Al fine di confermare l'identificazione a livello di specie, saranno eseguita l’identificazione biochimica tramite VITEK®2 (bioMérieux).

I profili di sensibilità antibiotica saranno valutati utilizzando il sistema automatizzato VITEK®2 (bioMérieux) e i breakpoint clinici EUCAST saranno utilizzati come criteri di interpretazione [www.eucast.org/clinical_breakpoints/]. Tutti i ceppi identificati saranno conservati a -80 ° C per successive analisi molecolari.

L'estrazione del DNA sarà effettuata da colture pure di *S. aureus* dopo 24 ore di incubazione a 37 ° C su agar Columbia + 5% di sangue di montone (bioMérieux) utilizzando il kit QIAamp DNA Mini (QIAGEN, Paesi Bassi) secondo specifiche del produttore.

Per determinare la potenziale virulenza dei ceppi di MRSA, verrà eseguito uno specifico test PCR per la presenza del gene Panton-Valentine Leukocidin (PVL) [Lina et al, 1999].

Durante il periodo di studio, il gene *mecA* e altri loci della cassetta SCCmec saranno caratterizzati seguendo diversi protocolli di amplificazione. Il protocollo suggerito da Oliveira e de Lancastre nel 2002 sarà utilizzato come primo test di screening e confermato dal metodo pubblicato da Zhang e colleghi nel 2005. Nuovi approcci sono stati recentemente sviluppati per evidenziare la presenza di SCC*mec*, di tipo VI, VII e VIII (Mileiriço *et al*, 2006; Higuchi *et al*, 2008; Zhang K *et al*, 2009, b), e saranno applicati per evidenziare la presenza di questi particolari tipi di SCC*mec*. Le analisi MLST saranno basate sulle sequenze nucleotidiche di 7 diversi geni housekeeping: gli isolati saranno definiti dagli alleli presenti nei sette loci (profilo allelico) e ad ogni profilo allelico univoco verrà assegnato un tipo di sequenza (ST). Gli isolati con la stessa ST avranno quindi sequenze identiche in tutti e sette i loci MLST e saranno considerati membri di un singolo clone (Enright *et al*, 2000).

<u>Metodi statistici</u>

Durante lo stesso periodo di 6 mesi valuteremo la variazione del FEV1 dei pazienti (% del predetto), lo stato nutrizionale (in termini di IMC), le riacutizzazioni polmonari (numero), i giorni trascorsi in ospedale e l'uso di antibiotici (giorni di trattamento) nel braccio attivo (A) e nel braccio osservazionale (B).

Gli obiettivi secondari saranno valutati ricorrendo a statistiche descrittive, a procedure statistiche parametriche e non parametriche per valutare eventuali differenze nelle variabili continue e categoriche tra i due gruppi, nonché a confronti nel periodo libero dall’infezione da MRSA. Il livello di significatività sarà fissato al 5%. I risultati per le variabili quantitative saranno espressi come media ± DS.
